# Clinical features of acute focal bacterial nephritis in adults

**DOI:** 10.1038/s41598-022-10809-5

**Published:** 2022-05-04

**Authors:** Sumin Jiao, Zhe Yan, Congqin Zhang, Juan Li, Jiaomei Zhu

**Affiliations:** Department of Nephrology, The Second Hospital of Hebei Medical University, Shijiazhuang, China

**Keywords:** Urinary tract infection, Bacterial infection

## Abstract

Acute focal bacterial nephritis (AFBN) is a localized bacterial infection of the kidneys presenting as an inflammatory mass that can develop into renal abscess. The current reports on AFBN mostly are among children and rarely described in adults. This study was aimed to analyze the clinical features of AFBN in adults and make a review for the disease to give the clinicians some clues to suspect and recognize it in adults. From January 2014 to December 2019, AFBN was diagnosed by contrast-enhanced computed tomography (CT) in 238 adults at the Department of Nephrology, the Second Hospital of Hebei Medical University, Shijiazhuang, China. We reviewed the clinical records of these patients and asked them about their post-discharge status via telephone follow-up. Of all the patients, 195 were female and 43 were male, the median age were 46.87 years. 86.13% presented with fever, 55.89% presented with lower urinary tract symptoms and 97.9% presented with pyuria. In renal ultrasonography, abdominal findings were seen only 22.69% patients. E.coli accounted for 74.73% of the isolated pathogen. After 4 weeks of treatment, the patients had no recurrence of symptoms. We recommend that when a patient presents clinically with acute pyelonephritis, but the fever persist longer after antimicrobial treatment (≥ 4 days in our study), AFBN should be suspected. For the diagnosis, contrast-enhanced CT is the “gold standard”, magnetic resonance imaging (MRI) may be a good option, but the ultrasonography is probably not satisfied. 3–4 weeks of antibiotic therapy may be appropriate for AFBN in adults.

## Introduction

Acute focal bacterial nephritis (AFBN), which is also known as acute lobar nephronia, is a radiological diagnosis that was first described in adults by Rosenfieldet al.^1^. AFBN appears as a single or multiple areas of focal bacterial infection in the renal parenchymal without liquefaction or abscess formation^2,3^. AFBN is considered to be the midpoint between acute pyelonephritis (APN) and renal abscess and represent an early stage of renal abscess. Patients with AFBN usually present with nonspecific symptoms, such as fever, flank or abdominal pain, urinary symptoms, pyuria, leukocytosis^4^, which is very similar with acute pyelonephritis, but radiologically, AFBN presents as renal mass, timely and adequate treatment could prevent unnecessary invasive surgical procedures and further progression to renal abscess or renal scarring, renal dysfunction^4–6^

AFBN becomes more common seen in China these years, but it is reported rarely in adults, most published studies have focused on children^6–11^. To give the clinicians some clues to suspect and recognize the AFBN in adults, we analyzed clinical data of 238 patients diagnosed with AFBN from January 2014 to December 2019 at the Department of Nephrology, the Second Hospital of Hebei Medical University.

## Methods

### Study population and methods

We retrospectively reviewed clinical data of 238 patients diagnosed with AFBN by contrast-enhanced computed tomography (CT) from January 2014 to December 2019 at the Department of nephrology, the second Hospital of Hebei Medical University, which is a tertiary medical centers located in the city of Shijiazhuang in north of China.

**Figure 1:**
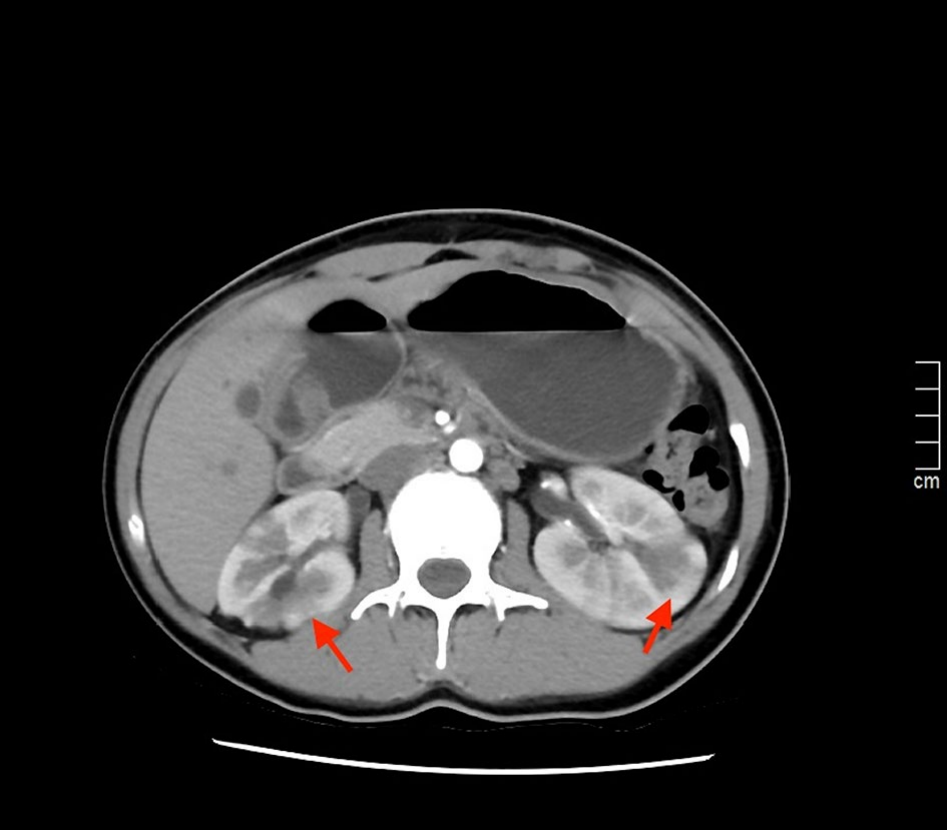
Typical findings for AFBN from contrast-enhanced CT. Images showing areas of wedge-shaped decreased enhancement in the left kidney and mass-like hypodense in right kidney (arrows).

AFBN diagnosis was made on the positive CT findings. CT examinations were performed using a GE spiral scanner (GE Medical Systems, Milwaukee, WI), using a 5-mm slice thickness and intervals was 5-mm as well. The most typical findings was a wedge-shaped decrease and/or mass-like hypodense lesions in nephrogenic density after injection of contrast medium^12,13^ (Fig. 1). The patients also performed ultrasonography (US). All patients received intravenous and oral antibiotics treatment for a total of 4 weeks after blood and urine cultures were taken. urine culture were considered to be positive if: at least 10^5^ colony-forming units (cfu)/mL pathogens from freshly voided midstream urine; at least one microorganism detected from the urine of suprapubic aspirations; or if > 10^4^ colony-forming units (cfu)/mL from urine sample obtained by transurethral catheterization^14–16^. Pyuria was defined as > 5 white blood cells (WBC) per microscopic high power field. All the statistical analyses were performed using SPSS 22.0.

### Ethics approval and consent to participate

This study was approved by the Ethics Committee of The Second Hospital of Hebei Medical University.

## Results

### Clinical characteristics of patients

Of all the patients, 195 were female and 43 were male, the ages of thepatients ranged from 18 to 82 years, with a median age of 46.87 years.More than half of the patients had risk factors, 68 (28.57%) with Diabetsmellitus, 41 (17.23%) with Urolithiasis,11 (4.6%) with Prostatic disease, 5 (2.1%) with immunodepression. The most common seen symptoms of the patients was fever, and then lower urinary tract symptoms, which include frequency and dysuria. Flank pain is also common seen in these patients, whereas nausea and/or vomiting only occasionally happened. For the treatment, second/third-generation cephalosporin, piperacillin tazobactam and carbapenem were about one-third each (Table 1).

**Table 1:** Clinical and laboratory findings of the patients with AFBN.

Variable	Value mean ± SD or n (%)
**Gender**	
Female n (%)	195 (81.93)
Male	43 (18.07)
Age (years)	46.87 ± 16.47
**Risk factors**	
Diabetes mellitus	68 (28.57)
Urolithiasis	41 (17.23)
Prostatic disease	11 (4.6)
Immunodepression	5 (2.1)
Repeated episodes of UTI	4 (1.68)
Pregnancy	4 (1.68)
**Symptoms**	
Fever	205 (86.13)
Absence of fever	33 (13.87)
Peak body temperature (°C)	39.31 ± 0.81
Lower urinary tract symptoms	133 (55.89)
Time to defervescence after antibiotic treatment(d)	3.94 ± 2.03
Flank or abdominal pain	107 (44.96)
Nausea and/or vomiting	6 (2.52)
**Antimicrobial agent**	
Second/third-generation cephalosporin	72 (30.25)
Piperacillin tazobactam	94 (39.5)
Carbapenem	75 (31.51)
Hospital stay (d)	19.0 ± 7.79

### Laboratory and imaging findings

All patients had blood and urine samples taken on admission. Laboratory findings showed an elevation of white blood cells (WBCs), neutrophil and procalcitonin. The incidence of pyuria was 97.9%. The glycosylated hemoglobin level was as high as 7.23. Positive urine culture was found in 91 (38.24%) of the 238 patients. The most common pathogen is E.coli, and then is enterococcus. Positive blood culture was observed only in 15 (6.3%), patients, E.coli is also the most common pathogen (86.67%), then is staphylococcus. The contrast-enhanced computed tomography (CT) scan indicated that 68(28.57%) patients had left AFBN, 54 (22.69%) had right AFBN, and 116 (48.74%) had bilateral AFBN. The ultrasonography (US) results showed nephromegaly in 52 (21.85%) patients, hypoechoic focal mass in only 2 (0.84%) patients (Table 2).

**Table 2:** Laboratory and image findings of patients with AFBN.

Variable	Value mean ± SD or n (%)
WBC (^~^10^9^/L)	11.96 ± 4.38
Neutrophils (%)	73.46 ± 13.43
Procalcitonin(ng/mL) median (inter-quartile range)	0.52 (0.18–1.34)
Blood hemoglobin (g/L)	116.36 ± 17.02
Serum albumin (g/L)	36.74 ± 5.61
HbA1c	7.23 ± 2.67
Pyuria	233 (97.9)
**Urine culture**	91 (38.24)
E.coli	68 (74.73)
Enterococcus	15 (16.48)
K pneumoniae	4 (4.4)
P aeruginosa	1 (1.1)
Flavobacterium odorata	2 (2.2)
Stenotrophomonas maltophilia	1 (1.1)
No isolatable organism	147 (61.76)
**Blood culture**	15 (6.3)
E.coli	13 (86.67)
Staphylococcus	2 (13.33)
No isolatable organism	208 (87.39)
Contrast-enhanced computed tomography (CT)	238 (100)
Bilateral	116 (48.74)
**Unilateral**	
Left	68 (28.57)
Right	54 (22.69)
Ultrasonography (US)	238 (100)
Nephromegaly	52 (21.85)
Hypoechoic area	2 (0.84)

## Discussion

Acute focal bacterial nephritis (AFBN) is considered as an intermediate form between acute pyelonephritis and renal abscess which belongs to upper urinary tract infection (UTI). If not diagnosed and treated timely and adequately, it may develop into renal abscess, thus leading to unnecessary invasive surgical procedures. But the diagnosis is difficult, as symptoms of AFBN are nonspecific and until now most of the reported cases are infants and children^17,18^. It is reported rarely in adults. To increase the awareness of AFBN in adults, we analyzed the clinical characteristics of 238 adult patients diagnosed with AFBN in our hospital in last 5 years.

UTI are more common in women than men because of the shorter urethra and the presence of antibacterial substances in male prostate fluid^19^. Consistent with this, women accounted for nearly 82% of all the subjects in our study. Most of our patients presented with nonspecific findings like fever (86.13%), lower urinary tract symptoms (55.89%) and flank or abdominal pain (44.96%), whereas nausea and/or vomiting only occasionally happened. Some patients presented only with fever or urinary symptoms. The symptoms in AFBN patients are of no difference with those in acute pyelonephritis patients^14^. Pyuria, leukocytosis, and elevated procalcitonin also were found in our patients. Yang, et al. found WBC counts and neutrophils counts were different between AFBN patients and non-AFBN patients, but they did not find the cut-off value between them^14^. High white blood cell counts, neutrophil counts and procalcitonin levels indicated that AFBN is a more severe renal parenchyma infection, consistent with this, patients often present with very high fever clinically, and the peak body temperatures in our study was 39.31 °C. It need more time to defervescence after antibiotic treatment, even the treatment is effective^8^. In children, fever lasting > 1.75 days after antibiotic treatment had a sensitivity of 92% and specificity of 79% for the detection of AFBN^17^. In our study, it took almost 4 days to bring the fever down after antibiotic treatment and thus lead to longer hospital stays, which is about 19 days in our study. By understanding the natural course of AFBN, we do not need to change antibiotics quickly because of the duration of the fever. Meanwhile, we should be suspicious of the possibility of AFBN for patients with UTI if the fever lasts for a long time.

E.coli was the leading cause of UTI^20^, previous reports found that E.coli represented over 90% of the microorganisms in AFBN children^14^. Consistent with this, E.coli accounted for 74.73% of pathogen in our study. The incidence of bacteremia in our study was only 15 (6.3%), this was similar with previous reports which was 5%^7^. The most common cause of bacteremia is also E.coli. E.coli showed high resistance to ampicillin and cotrimoxazole but low resistance to first and second generation cephalosporins or aminoglycosides, so, first and second generation cephalosporins are appropriate community-acquired UTI in children^14,21^. In our study, the use of second/third-generation cephalosporin, piperacillin tazobactam and carbapenem accounted for about one third each. It is suggested that the treatment of adult AFBN may be more difficult than that of children. The duration of antibiotic treatment for AFBN is not very clear until now, Cheng et al. suggested that a 3-week antibiotic treatment was sufficient while 2-week treatment could lead to relapse or persistent infection^7^. Therefore, timely and accurate differentiation of AFBN and APN can avoid inadequate treatment and prevent its progression to renal abscess. In our study, all patients received intravenous and oral antibiotics treatment for a total of 4 weeks. Although no follow-up laboratory results were available, patients reported no recurrence of symptoms during telephone follow-up.

In our study, diabetes mellitus, urolithiasis and prostatic disease were the top three underlying diseases, accounting for 28.57%, 17.23% and 4.6%, respectively. The average glycosylated hemoglobin level was as high as 7.23. However, Children with AFBN usually had urologic abnormalities like vesicoureteral reflux (VUR). The rate of VUR ranges from 22 to 44% in children with AFBN in other studies^8,12,14,22^, but the rate of VUR in non-AFBN children is similar with that in AFBN patients, suggesting that VUR is not a necessary factor for the development of AFBN^12,14^.

Previous studies had found that for the diagnosis of AFBN, ultrasonography (US) can detect nephromegaly with a sensitivity of 90.0% and specificity of 86.4%^23,24^. AFBN can also manifest as a focal renal mass with unclearly margins on US^3^.The focal renal mass may be hyper-, iso-, or hypoechoic depending on the time sequence of the lesion and the regression of the disease. In our study, of all the 238 AFBN patients, only 52 (21.85%) had nephromegaly and 2(0.84%) had hypoechoic focal mass, indicated that the sensitivity of US for AFBN diagnosis is probably not satisfactory, which was consistent with previous report^25^. Part of the reason for the different conclusions may be that the ultrasonic manifestations of AFBN are different in different periods^26^. Compared to US, contrast-enhanced computed tomography (CT) is currently recognized as the most sensitive and specific imaging modality for diagnosing and differentiating AFBN^12,22^. After contrast administration, AFBN typically appears as a wedge-shaped, non-enhancing, hypodense lesion^18^ and as mass-like hypodense lesions in the more severe form^27^. Studies about the sensitivity and accuracy of magnetic resonance imaging (MRI) in detecting AFBN had rarely been reported. But the few studies available suggested that MRI may be a good option for diagnosing AFBN^28,29^. Considering the absence of contrast agents and radiation exposure in MRI, further more studies are needed to confirm the role of MRI in the diagnosis of AFBN.

## Conclusion

In summary, when a patient presents clinically with acute pyelonephritis, but the fever persist longer after antimicrobial treatment (≥ 4 days in our study and ≥ 2 days in children^17^), AFBN should be suspected. For the diagnosis, contrast-enhanced computed tomography (CT) is the “gold standard”, magnetic resonance imaging (MRI) may be a good option, but the ultrasonography is probably not satisfied. The duration of antimicrobial treatment may need 3–4 weeks.

## Data Availability

The datasets used and/or analysed during the current study are available from the corresponding author on reasonable request.
